# Willingness to use video-game or gamified application-based interventions to improve or strengthen emotional regulation, mental health, and mental well-being

**DOI:** 10.1016/j.invent.2026.100950

**Published:** 2026-05-01

**Authors:** Germano Vera Cruz, Russell Pine, Terry Fleming, Magdalena Liberacka-Dwojak, Christelle Semaan, Elise Dan-Glauser, Monika Wiłkość-Dębczyńska, Yasser Khazaal

**Affiliations:** a Department of Psychology, UR 7273 CRP-CPO, University of Picardie Jules Verne, Amiens, France; b School of Health, Victoria University of Wellington, Wellington, New Zealand; c Department of Psychology, Kazimierz Wielki University, Bydgoszcz, Poland; d Institute of Psychology, Faculty of Social and Political Sciences, University of Lausanne, Lausanne, Switzerland; e Addiction Medicine, Department of Psychiatry, Lausanne University Hospital, Lausanne, Switzerland; f Research Centre, University Institute of Mental Health at Montreal and Department of Psychiatry and Addiction, Montreal University, Montreal, Canada

**Keywords:** Video games, Internet-based intervention, Emotional regulation, Mental health, Gaming disorder, Impulsivity, Digital well-being, User willingness

## Abstract

• **High overall openness:** Most respondents reported moderate to high willingness to use game-based interventions to improve emotion regulation, mental health, and well-being across diverse countries.
• **Two distinct user profiles identified:** Cluster analysis revealed a *high willingness* group (43%) and a *moderate willingness* group (57%), highlighting meaningful heterogeneity in receptivity.
• **Key predictors of high willingness:** Younger age (18–25), male gender, frequent gaming, gaming disorder symptoms, and specific emotion regulation difficulties significantly increased likelihood of high willingness.
• **Factors associated with lower willingness:** Higher impulsivity (low perseverance, high sensation seeking) and emotional unawareness or limited use of regulation strategies were linked to reduced openness.
• **One of the key findings:** Participants showed lower willingness to use game-based interventions preventively than reactively.

## Abstract (fr)

**Background:**

Game-based interventions show promise for mental health, yet limited research has examined individuals' willingness to engage and the factors shaping openness to these approaches.

**Objective:**

We aimed to (a) assess the extent to which individuals are willing to use game-based interventions to enhance emotional regulation, mental health, and mental well-being; (b) identify distinct participant profiles characterized by varying levels of willingness; and (c) examine socio-demographic, psychological, and game-related factors associated with this willingness.

**Methods:**

An online survey of 3745 adults aged 18–79 years from 48 countries assessed socio-demographic characteristics, gaming habits, impulsivity, emotional regulation difficulties, gaming disorder symptoms, and anxiety and depression symptoms.

**Results:**

Participants reported relatively high willingness to use game-based interventions for improving emotion regulation, improving mental health, and improving mental-wellbeing when feeling down, and more moderate willingness to use such interventions for improving mental wellbeing when feeling good (*M* = 3.25; 3.35; 3.26 and 2.93 respectively; 1–4 scale, where 1 is described as “Definitely would not” and 4 is “Definitely would”). Cluster analysis revealed two distinct profiles: a “high willingness” group (43%) and a “moderate willingness” group (57%). Logistic regression results indicated that being younger, male, and having higher gaming frequency, gaming disorder symptoms, and some emotional regulation difficulties dimensions significantly predicted membership in the “high willingness” group. In contrast, higher impulsivity and greater emotional unawareness or limited strategy use predicted a lower level of willingness.

**Conclusions:**

Most participants were open to digital mental health tools, with willingness shaped by individual traits, highlighting the value of tailored, culturally inclusive interventions for receptive subgroups, especially young adult gamers. One of the key findings of this study is that participants showed lower willingness to use game-based interventions preventively than reactively. This suggests preventive interventions may require distinct motivational mechanisms, with implications for designing strategies that enhance engagement during periods of well-being.

## 1 Introduction

Over the past two decades, video-games, smartphone applications (apps), and Internet-based interventions have emerged as potentially useful tools for psychological intervention and mental health promotion (Fleming et al., 2017; Lecomte et al., 2020; Linardon et al., 2024; Zhao et al., 2024). Such developments have the potential to support individuals' mental health in scalable and accessible ways (Andersson and Titov, 2014; Linardon et al., 2022).

A growing body of research has highlighted the therapeutic potential of digital games and gamified apps in fostering emotional awareness, self-regulation, behavior change, and well-being (Granic et al., 2014; Kato, 2017; Lin and Chang, 2025; Fleming et al., 2025; Domhardt et al., 2021). Serious games (full games designed for learning or behavior change) and gamified interventions (which add game elements to non-game activities to boost engagement and motivation) (Warsinsky et al., 2021) have been developed to target a wide range of psychological difficulties—including, among others, stress reduction, depression, anxiety, and emotion regulation—through mechanisms such as cognitive training, psychoeducation, behavioral activation, ecological momentary interventions and social connectedness (Ferguson and Olson, 2013; Li et al., 2022; Khazaal et al., 2015a; Pennou et al., 2023).

Despite these advances, the uptake and sustained engagement with digital and game-based mental health interventions remain a challenge, especially in naturalistic settings (rather than in controlled research settings) (Baumel et al., 2019a; Fleming et al., 2018). Previous studies suggest that willingness to engage with digital interventions is influenced by factors such as perceived usefulness, accessibility, privacy concerns, stigma, and individual differences in emotion regulation, impulsivity, severity of symptoms, and previous engagement in other digital and gaming tools (Deleuze et al., 2019; Diaz-Orueta et al., 2020; Kidman et al., 2024; Liu et al., 2025; Torous et al., 2018a; Vera Cruz et al., 2023a; Vera Cruz et al., 2023b). However, the relative importance of these determinants appears to vary considerably across individuals, populations, and usage contexts, limiting the generalizability of existing findings and complicating efforts to predict both interest or uptake and sustained engagement (Vera Cruz et al., 2023a; Vera Cruz et al., 2023b). Moreover, not all individuals perceive game-based tools as legitimate or effective means of improving psychological health (Kowert, 2020; Fleming et al., 2019). Understanding who is most willing to use such interventions in user-centered study design, and why, is essential for optimizing their design, dissemination, and clinical integration.

Emotion regulation—the ability to monitor, evaluate, and modify emotional responses—is a central component of mental health (Gross, 2015). Difficulties in emotion regulation have been linked to a range of psychopathologies, including mood and anxiety disorders, substance use, and behavioral addictions such as gaming disorder (Aldao et al., 2010; Kim et al., 2024; Stellern et al., 2023; Chrétien et al., 2025; Kraft et al., 2025). Given that many video games naturally engage emotional and cognitive regulation processes, game-based interventions may provide unique opportunities for training adaptive regulation strategies in engaging and motivational contexts (Villani et al., 2018; Pine et al., 2020). Yet, little is known about how individuals' emotional regulation capacities, impulsivity levels, and gaming habits relate to their openness toward using such tools for personal psychological growth and well-being enhancement.

Previous studies have tended to focus on the effectiveness of specific digital interventions or apps (Vera Cruz et al., 2023a; Firth et al., 2017; Lau et al., 2021; Etter and Khazaal, 2023) and adherence or retention in digital interventions once these have been begun. However, few have specifically examined willingness or interest in using video-game or gamified app-based interventions—a necessary precondition to ongoing engagement in them, and few examined interest across large, diverse samples, nor have they identified potential subgroups of users characterized by levels of willingness or psychological profiles. Specifically, examining willingness to engage in these tools and identifying distinctions in willingness and their correlates could inform the tailoring of digital interventions to user needs and increase their acceptability and effectiveness.

### 1.1 The present study

The present study aimed to address these gaps by examining willingness to use video-game or gamified application (apps)-based interventions (hereafter ‘game-based interventions’) for improving emotional regulation, mental health, and strength mental well-being. Using data from a large international sample of adults (n = 3745), we sought to (a) assess the overall extent to which participants were willing to engage with game-based interventions; (b) identify participant profiles characterized by different levels of willingness; and (c) explore the socio-demographic, psychological, and gaming-related factors associated with this willingness. Specifically, the study focused on individual characteristics such as gender, age, employment status, impulsivity, emotional regulation difficulties, and self-reported mental health problems, as well as gaming-related factors (gaming frequency and primary gaming device). By addressing these research questions, the study contributes to a better understanding of how individual differences shape openness to game-based and Internet-based mental health interventions, offering insights for future development and targeted implementation of digital therapeutics.

Research questions (RQs):

a) RQ1: To what extent are participants willing to use game-based interventions to improve or strengthen emotional regulation, mental health, and mental well-being?
b) RQ2: Can participants be clustered into distinct profiles (subgroups) according to their levels of willingness to use game-based interventions to develop or strengthen emotional regulation, mental health, and mental well-being?
c) RQ3: What individual characteristics (socio-demographic characteristics [gender, age, employment status], psychological characteristics [impulsivity, emotional regulation difficulties, game “addiction”, mental health and substance use diagnoses), and game habits [gaming frequency, main device use for gaming]) are associated with the willingness to use a game-based intervention for improving or strengthening emotion regulation, mental health, and mental well-being?

## 2 Methods

### 2.1 Participants

Inclusion criteria required participants to be aged 18 or older and fluent in English (the survey was in English). The recruitment targeted three subsamples: (1) young adult gamers (aged 18–25) who play at least 3 h weekly (n = 1700, 46.1%); (2) young adult gamers (aged 18–25, ≥3 h/week) with a history of depression or anxiety (n = 1312, 35.6%); and (3) a general population sample (aged 18–79) recruited without restrictions regarding gaming habits or mental health history (n = 673,18.3%). The sampling strategy led to an overrepresentation of women in the two first groups (1113 men vs 1899 women). This choice was based on the results of several previous studies (Vera Cruz et al., 2023b; Szinay et al., 2020; Lipschitz et al., 2020) showing that people who are highly involved in smartphone use and people who are concerned about their own mental health are more likely to use smartphone apps for mental health and well-being; and on the findings indicating that younger age groups and women are more likely to use such tools.

Overall, 3745 individuals from 48 countries completed an online survey. Most participants were from United Kingdom (UK; n = 1236, 33.0%), United States of America (USA; n = 1103, 29.5%), South Africa (n = 242, 6.5%), Canada (n = 181, 4.8%), Poland (n = 177, 4.7%) and the rest of the world (n = 806, 21.52%). For more details on the participants' country of residence, see Table S1, in Supplementary Material. The participants' ages ranged from 18 to 79 years old (mean [M] = 30.9, standard deviation [SD] = 12.8). The gender distribution was: male (n = 1490, 39.8%); female (n = 2255, 60.2%). The participants' employment status was as follow: unemployed and not seeking a job (n = 220, 6.9%); unemployed and seeking a job (n = 616, 19.2%); not in paid work (e.g., homework) (n = 361, 11.3%); due to start a new job within the next month (n = 55, 1.7%); part-time job (n = 693, 21.6%); full-time job (n = 1258, 39.3%).

### 2.2 Recruitment

The recruitment was conducted anonymously, using the online crowdsourcing platform Prolific (Prolific, 2023). Prolific has been described as having some advantages over other similar platforms, including that it is exclusively dedicated to research studies, and its participants are more ethnically and geographically diverse (Palan and Schitter, 2018; Peer et al., 2017).

### 2.3 Measures

The online survey included the following measures:

*Socio-demographic characteristics.* These characteristics consisted of gender (male, female), age, and employment status.

*Time using internet-based games*. The participants were required to indicate the number of typical hours spent playing Internet-based games on weekdays and weekends.

*Internet Game Disorder Symptoms*. The Internet Game Disorder Test (IGDT-10 (Király et al., 2017)), a unidimensional screening tool that assesses Internet Gaming Disorder as proposed in the fifth edition of the Diagnostic and Statistical Manual of Mental Disorders (DSM-5 (American Psychiatric Association, 2013)). Associated with each item, there is a 3-point response scale (Never–Often). In the present study, the Cronbach alpha coefficient (α) of this scale was 0.83.

*Emotional regulation difficulties*. The English version of the 6-item Difficulties in Emotional Regulation Scale Super Short Form (DERS-SSF; (Thuillard et al., 2025; Bjureberg et al., 2016; Gratz and Roemer, 2004)) was used. This instrument has 6 dimensions: (a) *non-acceptance of emotional response* (this refers to resistance or aversion to experiencing and accepting unpleasant emotions); (b) *difficulties in adopting goal-directed behaviors* (this refers to the challenges individuals may face when trying to engage in purposeful and intentional actions to achieve a specific objective or desired outcome); (c) *difficulties in controlling impulsive behaviors* (this occurs when individuals have difficulties in controlling impulsive behaviors triggered by emotions); (d) *lack of emotional awareness* (this refers to the difficulty in recognizing and identifying one's own emotions); (e) *limited access to emotion regulation strategies* (this refers to individuals who have fewer available strategies for effectively managing their emotions, resulting in difficulties in coping with emotional distress and regulating their mood); and (f) *lack of emotional clarity* (very similar to lack of emotional awareness, lack of emotional clarity refers, however, to the difficulties in understanding the differences between emotional states). There was one item for each dimension with a 5-point response scale for each (Almost never–Almost always). In the present study, the α of this scale was 0.72.

*Impulsivity* was assessed using the Short UPPS-P Impulsive Behavior Scale (Billieux et al., 2012). This is a measure of factors that could lead to impulsive behaviors. Participants respond to each item using a 4-point Likert scale: 1 (Strongly agree), 2 (Somewhat agree), 3 (Somewhat disagree), and 4 (Strongly disagree). *Positive Urgency* measures the tendency to act impulsively due to positive affect. In the present study, two items contribute to this score (e.g., “When I am in great mood, I tend to get into situations that could cause me problems”). *Negative Urgency* measures the tendency to act impulsively due to negative affect. Two items contribute to this score (e.g., “When I am upset, I often act without thinking”). *Lack of Premeditation* refers to the tendency to act rashly without first reflecting upon the decision to act. Two items contribute to this score (e.g., “I am not one of those people who blurt out things without thinking”). *Lack of Perseverance* involves a tendency not to complete projects. Two items contribute to this score (e.g., example of a reverse-coded item: “Unfinished tasks really bother me”). *Sensation Seeking* involves motivation to experience novelty. Two items contribute to this score (e.g., “I would like to learn to fly an airplane”). In the present study, the above dimensions of this scale had α of 0.73, 0.81, 0.65, and 0.78, respectively. Because this scale had two items in each dimensions, as recommended by (Eisinga et al., 2013), we also calculated the Spearman-Brown coefficient for each dimensions, as measure of reliability: 0.73, 0.81, 0.64, and 0.78, respectively.

*Mental health problems* was assessed using the 4-item Patient Health Questionnaire for Anxiety and Depression (PHQ-4 (Löwe et al., 2010)) which is designed to assess: *anxiety* (“Over the last two weeks, how often have you been bothered by the following problems? Feeling nervous, anxious or on edge”); and *depression* (“Over the last two weeks, how often have you been bothered by the following problems? Feeling down, depressed or hopeless) with response options of a 4-point scale (Not at all–Nearly everyday). In the present study, the two dimensions of this scale had α of: anxiety = 0.87; depression = 0.83. In addition, participants self-reported if they had sought help for a mental health problem and if they had ever been diagnosed with a mental health or substance use problem.

*Usefulness and willingness to use game-based interventions.* Participants were asked to indicate their level of agreement that “video-game, apps, or gamified apps-based interventions could be useful for improving mental health” on a 1 (Strongly disagree) to 5 (Strongly disagree) response scale. Finally, four items inquired the participants' willingness to use video games for (a) improving emotional regulation difficulties; (b) improving mental health; (c) strengthening mental well-being when feeling down; and (d) maintaining/strengthening mental well-being when already feeling good. A 4-point scale (definitely would not, probably would not, probably would, definitely would) was applied, with high scores indicating higher levels of willingness.

It must be noted that, although the four specific items described above have not been previously validated as a standardized scale, their content validity was carefully considered. Each item was designed to capture a distinct and theoretically meaningful dimension of willingness to use game-based interventions (i.e., emotional regulation, mental health improvement, and well-being enhancement in both negative and positive states), thereby ensuring adequate conceptual coverage of the construct.

### 2.4 Ethics

Participants gave digital informed consent for participating in the study and completing the survey. Participation was voluntary and restricted to those aged ≥18. All data was anonymously collected. The study was conducted in accordance with the Declaration of Helsinki — Ethical Principles for Medical Research Involving Human Subjects (World Medical Association (WMA), 1964). Ethical approval for the research project (no. 8/18.11.2025) was obtained from the Ethics Committee of the Kazimierz Wielki University in Bydgoszcz.

### 2.5 Data analytics

To address RQ1, we conducted descriptive statistics (mean [M], standard deviation [SD], and frequencies) on all variables included in the study.

To address RQ2, we performed K-mean cluster analysis (Everitt et al., 2011) using the four measures of willingness to use video games (improving emotional regulation difficulties; improving mental health; strengthening mental well-being when feeling down; maintaining/strengthening mental well-being when already feeling good). Cluster analysis is particularly appropriate for addressing this RQ, as it enables the identification of homogeneous subgroups of participants based on their patterns of responses regarding willingness to use game-based interventions. Rather than assuming a uniform distribution of attitudes across the sample, this person-centered approach allows for the detection of distinct profiles that may differ meaningfully in their levels of willingness or reluctance. Such profiling can provide deeper insights into variability in engagement tendencies and inform the development of tailored intervention strategies targeting specific subgroups. Compared to a median split, which imposes an arbitrary dichotomy and reduces variability, cluster analysis preserves information, identifies data-driven subgroups, captures multidimensional patterns, and reveals multiple meaningful profiles, offering a more accurate and practically useful approach for understanding heterogeneous willingness toward game-based interventions.

Hopkins statistic value (H = 0.75) indicates a strong clustering tendency, thereby supporting the appropriateness of applying cluster analysis to our data (Lawson and Jurs, 1990; Drab and Daszykowski, 2014). This reduces the likelihood that the observed structure is merely an artefact of scale properties. In addition, the use of the Gap statistics (Everitt et al., 2011) supported the selection of the two-cluster solution based on model fit rather than assumption (see Fig. S1 and S2, on Supplementary Material). Importantly, inspection of cluster centroids (see Tables S3 and S4, on Supplementary Material) revealed systematic differences across dimensions, which would be obscured by a unidimensional dichotomization.

Third, to address RQ3, we create a binomial logistic regression model (*LG Model-1*) including the participants' socio-demographic characteristics (gender, age, employment status), psychological characteristics (scores of impulsivity, emotional regulation difficulties, game addiction, mental health and well-being variables), mental health and substance use diagnoses as predictors, and the clusters of willingness to use video-game and Internet-based interventions to improve emotional regulation, mental health and well-being, as outcomes. This was based on the results of the previous analyses and included two clusters: cluster-1 (high willingness subgroup) and cluster-2 (moderate willing subgroup). A Pearson correlation matrix conducted prior to modeling showed no multicollinearity, as no coefficients exceeded 0.70 (Vatcheva et al., 2016). The model was designed to predict membership in the “high willingness group” (coded as 1), using the “moderate willingness group” (coded as 0) as the reference class.

Regarding the *LR Model-1*, it is important to note that the variables related to the sampling design (“group of participants”) were operationalized as two dichotomous predictors: (a) gamers aged 18–25 with a history of depression/anxiety versus gamers aged 18–25 without such a history; and (b) the general population aged 18–79 versus gamers aged 18–25. In addition, participants' chronological age and gaming frequency were included as continuous variables, reflecting their raw measurement and not as grouping variables derived from the recruitment strategy. Also, it must be noted that the logistic regression model included two distinct depression/anxiety variables: (a) a dichotomous indicator of lifetime history of depressive/anxiety episodes (linked to the sampling strategy and group membership), and (b) current depressive symptoms measured using the PHQ, treated as an ordinal predictor. Finally, we conducted sensitivity analyses by estimating a second logistic regression model (*LG Model-2*) that excluded the two sampling-related participant group predictor variables. This approach enabled a comparison of the findings across the two models.

Descriptive analysis was conducted using IBM SPSS Statistics (2025). Cluster analysis was performed using R statistical programming (package “snowCluster” and “factoextra” (Seol, 2024; Kassambara and Mundt, 2020; R Core Team, 2022)). Logistic regression modeling was conducted using Jamovi software (Version 2.4 (The Jamovi Project, 2023)).

## 3 Results

### 3.1 Willingness to use video-game and gamified apps to improve or strengthen emotional regulation, mental health, and wellbeing (RQ1)

Table 1 presents the descriptive results of the participants across the main measures administered. Table S2, in Supplementary Material, displays detailed descriptive statistics for all the study variables. As shown in Table 1, participants indicated agreement (4.06 points, on a 1 (Strongly disagree) to 5 (Strongly disagree) scale) that “video-game, apps, or gamified apps-based interventions could be useful for improving mental health”. In addition, Table 1, shows that, on a 1 (Definitely would not) to 4 (Definitely would) point scale, participants indicated moderate to high willingness to use game-based interventions to improve/strengthen emotional regulation (*M* = 3.25), and to improve mental health (*M* = 3.35), and to develop/strengthen mental well-being when feeling down (*M* = 3.26), and to maintain/strengthen mental well-being when feeling good (*M* = 2.93) — taken into account the midpoint (2 points) of the 1–4 scale.

**Table 1:** Descriptive statistics for the study main variables.

Variable	N	Min–Max	Mean	SD	Frequency
Group of participants					Gamers aged 18–25 = 1700 (46.1%); Gamers aged 18–25 with depression and/or anxiety episodes = 1312 (35.6%); General population of non-gamers = 673 (18.3%).
Sociodemographics					
Age	3745	18–79	30.94	12.76	
Sex					Male = 1490 (39.80%) Female = 2255 (60.20%)
Employment status	3745				Full-time job = 1258 (39.3%). Part-time job = 693 (21.6%); Unemployed (and job seeking) = 616 (19.2%); Not in paid work (e.g. homework, retired or disabled) = 361 (11.3%); Due to start a new job within the next month = 55 (1.7%); Other = 220 (6.9%).
Gaming frequency					
Daily hours playing video-game	3060	2–5	3.64	1.16	
Hours playing video-games (Weekdays)	3685	0–120	13.50	12.26	
Hours playing video-games (Weekends)	3742	0–60	7.93	6.31	
Internet Gaming Disorder Test-10 (IGDT-10)					
IGDT total mean score	3685	1–3	1.63	0.39	
Impulsive Behavior Scale (UPPS-P)					
UPPS-P mean for urgency	3678	1–5	2.43	0.934	
UPPS-P mean for premeditation	3678	1–5	2.470	0.91	
UPPS-P mean for perseverance	3678	1–5	2.670	0.88	
UPPS-P mean for sensation seeking	3678	1–5	2.774	1.02	
UPPS-P total mean score	3678	1–5	2.90	0.52	
Patient Health Questionnaire for Anxiety and Depression (PHQ-4)					
PHQ mean for anxiety	3674	1–4	2.24	0.94	
PHQ mean for depression	3674	1–4	2.09	0.90	
PHQ total mean score	3674	1–4	2.17	0.85	
Difficulties in Emotion Regulation Scale Super Short Form (DERS-F-SSF-6)					
DERS-Item1 (awareness)	3666	1–5	2.41	1.06	
DERS-Item2 (clarity)	3666	1–5	2.37	1.13	
DERS-Item3 (goals)	3666	1–5	3.29	1.22	
DERS-Item4 (not acceptance)	3666	1–5	2.56	1.28	
DERS-Item5 (strategies)	3666	1–5	2.32	1.14	
DERS-Item6 (impulse)	3666	1–5	1.95	1.07	
DERS total mean score	3666	1–5	2.58	0.773	
Usefulness and willingness to use game/app/program to improve wellness					
Level of agreeing that a video-game, gamified app or Internet-based program could be useful for improving mental health	3662	1–5	4.06	0.77	
Willingness to use video game, app, and online-based interventions to develop or strengthen mental well-being?	3661	1–4	3.35	0.70	
Willingness to use video-game, app, and Internet-based interventions to develop or strengthen emotional regulation	3663	1–4	3.25	0.75	
Willingness to use video-game and Internet-based interventions to develop or strengthen mental health when feeling down	3656	1–4	3.26	0.74	
Willingness to use video-game or gamified apps-based interventions to maintain or strengthen mental well-being when feeling good	3655	1–4	2.93	0.97	
Mental health problems					
Diagnosed with a mental health problem	3745				No = 1992 (54.4%); Yes = 1672 (45.6%).
Diagnosed substance use addiction	3745				No = 3477 (94.9%); Yes = 188 (5.1%).

Note. N = number of participants. Mix–Max = minimum–maximum. SD = standard deviation.

### 3.2 Distinct profiles of participants (subgroups) according to their levels of willingness to use video-game and gamified apps-based interventions to develop or strengthen emotional regulation, mental health, and mental well-being (RQ2)

**Fig. 1:**
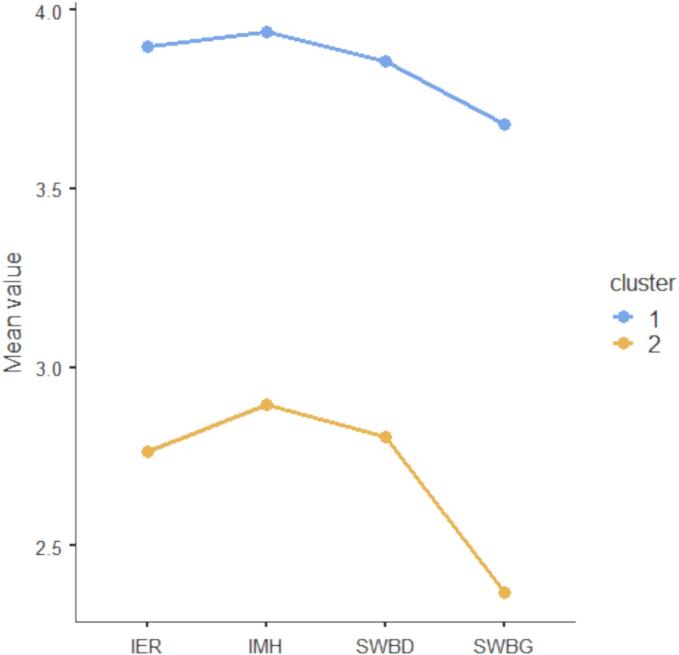
Graphic of means across clusters. On the vertical axis, the mean values for the four variables included in the cluster analysis (4-point scale). On the horizontal axis, the four variables included in the cluster analysis: IER = willingness to use video-game and gamified apps-based interventions to improve emotional regulation; IMH = willingness to use video-game and gamified apps-based interventions to improve mental health; SWBD = willingness to use video-game and gamified apps-based interventions to improve mental well-being when feeling down; SWBG = willingness to use video-game and gamified apps-based interventions to strength mental well-being when already feeling good. Cluster-1 = high levels of willingness to use video-game, apps and Internet-based interventions to improve psychological health. Cluster-2 = moderate levels of willingness to use video-game and gamified apps-based interventions to improve psychological health.

Cluster analysis yields two clearly distinct clusters. As can be seen in Fig. 1 (displaying the variables' mean values across the two clusters), cluster-1 (n = 1564, 43%; total participants = 3637, 100%) indicates that the participants of this subgroup had relatively high levels of willingness to use game-based interventions to improve psychological health (that is: improve emotional regulation difficulties, improve mental health, strength mental well-being when feeling down, maintain/strength mental well-being when already feeling good). Conversely, cluster-2 (n = 2073, 57%; total participants = 3637, 100%) suggests that the participants of this subgroup have moderate levels of willingness to use game-based interventions to improve psychological health.

Table S3, in the Supplementary Material, presents the statistics related to the cluster centroids. Table S4, in the Supplementary Materials, presents the descriptive statistics (means and standard deviations) for willingness to use game-based interventions across clusters, along with inferential statistics (independent-samples *t*-tests) comparing the mean differences between the two clusters. Fig. S2, in the Supplementary Material, shows a graphic representation of the chosen two-cluster solution.

### 3.3 Variables predicting cluster belonging (RQ3)

Table 2 shows the binomial logistic regression model of predictor-outcome relationships. The following are the model performance statistics: Accuracy = 0.76, indicating 76% good prediction; Pseudo R-Square (Nagelkerke's R^2^) = 0.32, indicating that the model's predictor variables account for 32% of the criterion (outcome) variable's change (Menard, 2002).

**Table 2:** Relationships between the predictor variables and the clusters: binomial logistic regression results.

Predictor variable	N	Estimate (*b*)	SE	*Z*	*p*	OR	OR 95%CI	
							Lower	Upper
Group of participants								
*18–25 years old gamers with history of depression/anxiety episodes* VS. *18–25 years old gamers who did not report history of depression/anxiety*		0.511	0.074	6.84	<0.001	1.667	1.440	1.930
*18–79 years old general population* VS. *18–25 years old gamers*		−0.713	0.101	−7.04	<0.001	0.490	0.402	0.598
Sociodemographics								
Age	3745	0.012	0.006	1.92	0.055	1.013	1.000	1.025
Sex	3745							
*Male* VS. *Female*		0.444	0.123	3.58	<0.001	1.559	1.223	1.987
Employment status	3745							
*Unemployed (and job seeking)* VS. *Other*		−0.055	0.175	−0.31	0.752	0.946	0.671	1.334
*Not in paid work (*e.g.*, ‘homework’, ‘retired or disabled’)* VS *Other*		0.189	0.211	0.89	0.372	1.208	0.798	1.830
*Due to start a new job within the next month* VS. *Other*		0.315	0.331	0.95	0.341	1.371	0.716	2.625
*Part-time job* VS. *Other*		0.185	0.174	1.06	0.288	1.204	0.855	1.697
*Full-time job* VS. *Other*		0.065	0.168	0.39	0.695	1.068	0.768	1.485
Gaming use frequency								
Daily hours playing video-game	3060	−0.005	0.038	−0.14	0.884	0.994	0.923	1.071
Weekly (Weekday?) hours playing video-game	3685	0.009	0.004	2.02	0.043	1.009	1.000	1.018
Week-end hours playing video-game	3742	−0.000	0.008	−0.06	0.945	0.999	0.983	1.016
Internet Gaming Disorder Test-10 (IGDT-10)								
IGDT mean score	3685	0.559	0.130	4.28	<0.001	1.750	1.355	2.262
Impulsive Behavior Scale (UPPS-P)								
UPPS-P mean for urgency	3678	0.045	0.057	0.79	0.427	1.047	0.935	1.172
UPPS-P mean for lack premeditation	3678	−0.095	0.052	−1.82	0.069	0.909	0.821	1.007
UPPS-P mean for lack perseverance	3678	−0.164	0.049	−3.31	<0.001	0.848	0.769	0.935
UPPS-P mean for sensation seeking	3678	−0.175	0.043	−4.05	<0.001	0.839	0.771	0.913
Patient Health Questionnaire for Anxiety and Depression (PHQ-4)								
PHQ mean for anxiety	3674	0.020	0.064	0.31	0.749	1.021	0.900	1.158
PHQ mean for depression	3674	0.0010	0.005	2.03	0.0429	1.010	1.000	1.020
Difficulties in Emotion Regulation Scale Super Short Form (DERS-SSF)								
DERS-Item1 (awareness)	3666	−0.225	0.042	−5.32	<0.001	0.798	0.735	0.867
DERS-Item2 (clarity)	3666	0.032	0.043	0.74	0.458	1.033	0.948	1.126
DERS-Item3 (goals)	3666	0.103	0.043	2.40	0.016	1.110	1.019	1.208
DERS-Item4 (not acceptance)	3666	0.111	0.038	2.89	0.004	1.118	1.036	1.205
DERS-Item5 (strategies)	3666	−0.098	0.047	−2.07	0.038	0.906	0.825	0.995
DERS-Item6 (impulse)	3666	−0.003	0.047	−0.08	0.935	0.996	0.907	1.094
Psychopathology diagnosis								
Diagnosed with mental health problem	3745							
Yes VS. no		0.12426	0.09644	1.2885	0.198	1.132	0.937	1.368
Diagnosed substance use addiction	3745							
Yes VS. no		0.18929	0.18698	1.0124	0.311	1.208	0.838	1.743

*Note*. Estimates represent the log odds of “Clusters = 1” vs. “Clusters = 0”. N = number of participants. *b* = beta coefficient. *Z* = *z*-values. *p* = *p*-values (significance at <0.05). OR = odd ratio. CI = interval of confidence. VS = versus.

The odds ratio (OR) value is the *b* (beta) coefficient exponent. To interpret it, one must consider that, for example, an OR = 2.30 means that there are 2.30 times the odds of belonging to the target subgroup (high willingness group) compared to the other subgroup (moderate willingness group); an OR = 1.75 means that there is an 75% increase in odds; and an OR = 0.84 means that there is a 16% decrease in odds.

As shown in Table 2, 18–25 years old gamers with a history of depression/anxiety were significantly more likely to belong to the cluster-1 (reporting higher willingness) than 18–25 years old gamers who did not report a history of depression/anxiety (OR = 1.66, *p* < 0.001). Furthermore, 18–25 years old gamers with a history of depression/anxiety were more likely to belong to cluster-1 than participants from 18 to 79 years old general population; similarly, 18–25 years old gamers who did not report history of depression/anxiety were more likely to belong to cluster-1 when compared with participants from 18 to 79 years old general population. Male participants were more likely to belong to cluster-1 than females (OR = 1.55, *p* < 0.001).

In terms of statistical significance, participants were also more likely to belong to cluster-1 (high willingness) if they spent more time playing video-games during the week-end (OR = 1.01, *p* = 0.043), had high scores on the IGD-10 (OR = 1.75, *p* < 0.001), had high scores on the non-acceptance of emotional response (OR = 1.11, *p* = 0.016) or difficulties in adopting goal-directed behaviors dimensions of the difficulties in emotional regulation scale (OR = 1.11, *p* = 0.004), had high scores on the depression dimension of the PHQ (OR = 1.01, *p* = 0.042).

In contrast, participants who had high scores of impulsivity on lack of perseverance and the sensation seeking dimensions were statistically more likely to belong to cluster-2. Participants with high scores on emotional unawareness or limited strategy use dimensions of the difficulties in emotional regulation scale were also more likely to belong to cluster-2 (respectively, OR = 0.79, *p* < 0.001; OR = 0.90, *p* = 0.038).

Finally, sensitive analyses — *LR Model-2* (logistic regression model without the two modeled group participants' predictor variable; see Table S5 in Supplementary Material) yield the same results as the *LR Model-1* (results of which we reported above).

## 4 Discussion

### 4.1 Level of willingness to use game-based interventions

The present study investigated the willingness of individuals to use game-based interventions aimed at improving emotional regulation, mental health, and well-being across a large international sample. Overall, findings revealed a relatively high willingness to engage with such interventions, particularly for mental health problems, emotion regulation, and for improving wellbeing when feeling down (with slightly lower willingness to use game-based interventions for mental wellbeing when already feeling good). Participants demonstrated stronger agreement with statements emphasizing the usefulness of video and gamified app-based tools for improving mental health and emotion regulation, suggesting that such tools are increasingly perceived as legitimate means of promoting psychological health. These findings align with previous research showing growing acceptance of digital mental health tools, particularly among younger adults and those already familiar with gaming environments (Firth et al., 2017; Lau et al., 2021).

### 4.2 Profiles of willingness

Cluster analysis identified two distinct participant profiles. The first cluster, representing approximately 43% of respondents, consisted of individuals with a relatively high willingness to use digital and game-based interventions. The second cluster (57%) reflected moderate willingness and more reserved attitudes toward such technologies. This dichotomy mirrors earlier findings that individuals differ not only in their access to or familiarity with digital tools but also in their motivation and perceived relevance of these tools for self-care (Linardon et al., 2024). Importantly, the present study extends prior work by empirically identifying these profiles within a heterogeneous global sample, rather than assuming homogeneous user receptivity.

It is important to highlight the fact that this study clearly identifies two major clusters: one group with relatively high willingness and openness to gaming and gamified-apps related interventions, and another group with moderate willingness and having hesitant or ambivalent attitudes toward such technologies. This dichotomy is supported by empirical findings showing that engagement and receptivity are not homogeneous, but instead vary according to individual motivation, perceived relevance, and capacity to invest effort in digital interventions (Zainal et al., 2025; Smail-Crevier et al., 2019; Nowels et al., 2024). In addition, these studies have shown that demographic and psychosocial factors—such as age, gender, education, socioeconomic status, prior mental health experience, and capacity to invest effort—are associated with engagement levels. For example, individuals with lower capacity to invest effort are more likely to prefer digital self-help tools over traditional psychotherapy, while those with higher capacity favor professional-led interventions (Zainal et al., 2025). Engagement clusters also differ in behavioral, cognitive, and affective engagement, with some users achieving significant symptom improvement despite lower behavioral engagement (Smail-Crevier et al., 2019). These findings highlight the need for precision approaches in digital mental health, emphasizing flexible, user-centered design to accommodate varying engagement profiles and preferences across diverse populations (Smail-Crevier et al., 2019; Nowels et al., 2024; Escoffery, 2018). In sum, these findings suggest people need options and that digital mental health interventions and gamification are not one size fits all.

Finally, according to the cluster analysis results, participants had significantly lower willingness to use game-based interventions for maintaining well-being when already feeling good (preventive use) compared to improving mental health when feeling down (reactive use). This striking finding might mean that “preventive” interventions (used during periods of well-being) may require different motivational mechanisms than “reactive” or “rescue” interventions (used during periods of feeling down). In addition, this pattern aligns with health behavior models, particularly the Health Belief Model, which posits that perceived susceptibility and severity of a health threat are key motivators for health behavior change (Naslund et al., 2017). In contrast, during periods of well-being, the perceived threat is low, and the benefits of preventive engagement may seem abstract or distant, reducing motivation despite potential long-term value. This distinction has important implications for intervention design and implementation.

### 4.3 Predictors of willingness

The logistic regression model provided insight into the individual characteristics shaping willingness. Participants who reported a history of depression and or anxiety episodes were significantly more likely to belong to the “high willingness” cluster. These findings should be distinguished from those concerning the association between participants' current experiences of anxiety and depressive mood, as measured by PHQ, and the outcome variable. Specifically, anxiety was not statistically associated with the outcome, whereas depressive mood was significantly associated; however, its effect size was very small (OR = 1.01). Meanwhile, previous studies results have shown higher adoption rates of mental-health-related apps among individuals with elevated distress or reduced access to traditional care (Baumel et al., 2019a; Poppelaars et al., 2018), which is partially consistent with the findings of the present study. This willingness is important. In contrast to findings of ‘help negation’, whereby those with higher mental health needs are less likely to consider that interventions will help them and are forestalled from help seeking, perhaps as part of feelings of hopelessness or being overwhelmed (Wilson, 2010; Wilson and Deane, 2012; Turner et al., 2024), our findings suggest those facing mental health challenges may be more open to game-based interventions for mental health. Help negation appears particularly affect young men (Wilson, 2010; Wilson and Deane, 2012; Turner et al., 2024), hence our finding of high willingness to use game-based interventions among distressed young males is noteworthy. Game-based interventions may reduce care barriers access including perceived stigma by framing mental health support within a familiar leisure activity; they offer anonymity and autonomy that align with preferences for self-reliance; and they leverage existing gaming habits and digital self-efficacy among people (Sheikh et al., 2025). This represents a significant opportunity to engage a population that is notoriously difficult to reach through conventional services.

Younger participants (particularly aged 18–25) and male participants were also more likely to express willingness to use game-based interventions than their older or female counterparts. Gender differences may reflect gendered differences in gaming familiarity and self-efficacy with technology. The findings contrast with previous studies showing that females are more willing to use smartphone apps or internet-based interventions for their mental health compared to male. Indeed, multiple recent meta-analyses and large-scale studies consistently demonstrate that women have higher initial uptake, usage, and completion rates for digital mental health interventions than men (Zainal et al., 2025; Smail-Crevier et al., 2019; Nowels et al., 2024; Escoffery, 2018; Knipe et al., 2025; Zhang et al., 2025; Jagemann et al., 2024). Women also report greater willingness to use internet-based resources for mental health information and support (Zainal et al., 2025; Smail-Crevier et al., 2019; Nowels et al., 2024; Escoffery, 2018; Knipe et al., 2025; Zhang et al., 2025; Jagemann et al., 2024). Men tend to engage more frequently with video-games (Entertainment Software Association, 2024), potentially increasing comfort with gamified therapeutic formats. However, the finding also underscores the need to design different tools to appeal to different groups — by understanding and segmenting interest groups, rather than offering homogenous responses, it may be possible to appeal more to different groups (Fleming et al., 2023).

Gaming frequency and more severe symptoms of gaming disorder were both positively associated with willingness to use game and Internet-based tools for mental health. This association may appear paradoxical, as gaming disorder involves maladaptive patterns of use. Nevertheless, it may indicate that individuals who game frequently recognize the immersive and emotional potential of games and may be more open to using similar platforms for self-regulation. Such openness could provide an opportunity to channel gaming engagement toward health-related, purpose-driven applications (Kowert, 2020).

Regarding psychological predictors, impulsivity dimensions showed divergent relationships. High lack of perseverance and high level of sensation seeking predicted lower willingness, suggesting that individuals who are either easily bored, resistant to structured engagement, or afraid of challenges, or task-related difficulties may perceive game-based interventions as less appealing. The urgency dimensions did not discriminate between groups, suggesting that the high urgency groups, with high needs for specific interventions, may be open to such support (Billieux et al., 2021). Conversely, specific emotion regulation difficulties—particularly non-acceptance of emotions and difficulty maintaining goal-directed behavior under distress—were associated with higher willingness; whereas other difficulties, like low awareness and limited access to strategies were associated with lower willingness. The pattern of findings suggests that willingness to engage with game-based interventions is not driven by emotion regulation difficulties per se, but by how these difficulties are cognitively represented and experienced. Higher non-acceptance of emotions and greater disruption of goal-directed behavior under distress may reflect situations in which emotions are both salient and functionally impairing, thereby motivating individuals to seek external regulation support. In contrast, low emotional awareness and limited perceived access to regulation strategies were associated with unwillingness, potentially reflecting reduced insight into emotional processes or low perceived self-efficacy regarding emotion regulation.

### 4.4 Theoretical and practical implications

The present findings highlight the complex interplay between gaming habits, emotional functioning, and openness to gaming and gamified apps for mental health needs. They support the notion that emotional regulation difficulties can serve both as a motivator and a barrier to digital mental health engagement, depending on their nature and level of self-awareness. From a theoretical standpoint, these results align with Gross's (2015) process model of emotion regulation, which emphasizes awareness and acceptance as prerequisites for adaptive regulation strategies.

From a practical perspective, tailoring digital interventions to user profiles appears crucial. Developers and clinicians may enhance uptake by customizing intervention content and aesthetics to match users' psychological needs, gaming familiarity, and motivational tendencies. Involving end users and people with lived experience may possibly increase willingness for use and potential engagement in the interventions (Huot-Lavoie et al., 2025; Fleming et al., 2016). For instance, incorporating personalized feedback, goal-setting, and emotionally resonant narratives could increase perceived relevance and emotional engagement. Furthermore, targeting younger users and those with existing gaming experience could be an effective starting point for large-scale dissemination, while parallel strategies should be developed to engage older adults and those less digitally inclined. Our findings highlight that user behavior and preferences are important. While ‘user segmentation’ is not new in game design, mental health interventions are often targeted to all youth or adults with specific mental health problems. Improved targeting based on more nuanced user characteristics and willingness to try a specific type of intervention is an important opportunity in improving the appeal of mental health interventions. In addition, providing options for both highly gamified and minimally gamified experiences, or integrating educational content that demystifies digital interventions, may help address users' reservations about engaging with such tools.

From a clinical implementation perspective, the high willingness observed among individuals with prior depression and anxiety episodes suggests an important opportunity to integrate game-based interventions into existing mental health care pathways (Panarello et al., 2025; Graham et al., 2020; Khazaal et al., 2015b). These interventions may serve valuable roles within stepped-care models—for example, as low-intensity first-line options for individuals with mild-to-moderate symptoms, as adjuncts to ongoing psychotherapy or pharmacotherapy to enhance skill practice between sessions, or as maintenance tools following formal treatment to prevent relapse. However, successful integration will require clear clinical guidance (Panarello et al., 2025; Graham et al., 2020; Khazaal et al., 2015b).

### 4.5 Limitations

Despite its strengths—such as the large, diverse international sample and the integration of behavioral, psychological, and demographic predictors—the study has several limitations. First, the cross-sectional design precludes causal inference; while psychological variables were associated with willingness, the directionality of these relationships remains uncertain. Second, all measures relied on self-report instruments, which may be affected by social desirability and recall bias. In addition, although these specific items have not been previously validated as a standardized scale, their content validity was carefully considered. Each item was designed to capture a distinct and theoretically meaningful dimension of willingness to use game-based interventions (i.e., emotional regulation, mental health improvement, and well-being enhancement in both negative and positive states), thereby ensuring adequate conceptual coverage of the construct. Also, the four single-items used to measure the level of the participants' willingness to use gamified interventions were not subjected to test–retest reliability and other psychometric indicators (e.g., internal consistency) among this study target population, which would strengthen confidence in these measures. We acknowledge this as a limitation. Third, although the study included participants from multiple countries, recruitment through an online crowdsourcing platform (Prolific) may have attracted individuals with greater digital literacy or interest in gaming, potentially limiting generalizability to less tech-savvy populations. Fourth, the study did not assess cultural or contextual factors—such as stigma toward mental health treatment or local gaming norms—that may influence willingness differently across regions. Fifth, it must be noted that some predictor variables, while significantly related to the outcome according to the *p*-value (e.g., weekend gaming frequency and depressive mood as measured by PHQ) should be interpreted with caution. Although statistically significant, their effect sizes (as indicated by the odds ratios) were very small, indicating limited practical significance (Rosen and DeMaria, 2012). This suggests that the observed associations may have minimal real-world impact, independent of sample size. Overall, these findings imply that willingness to engage in game-based interventions is likely influenced by a constellation of small, distributed factors rather than by strong individual-level predictors. Sixth, while the regression model demonstrated satisfactory predictive accuracy, the pseudo-R^2^ value suggests that unmeasured variables (e.g., attitudes toward technology, personality traits, or previous exposure to digital therapy) also contribute to willingness and should be considered in future research.

Finally, the findings from the present study must be interpreted in light of the well-established distinction between self-reported willingness and actual engagement behavior. While willingness reflects a favorable attitudinal disposition toward game-based interventions, it does not necessarily translate into their initiation, sustained use, or adherence over time. Consistent with prior research (Smoktunowicz et al., 2023), intention–behavior gaps are frequently observed in the context of digital and internet-based interventions, where initial motivation often declines once individuals are confronted with practical, contextual, or experiential barriers.

### 4.6 Future research directions

Future research should build on these findings by employing longitudinal and experimental designs to track actual engagement, adherence, and clinical outcomes over time, thereby addressing the well-documented gap between self-reported willingness and real-world behavior. The development and psychometric validation of comprehensive, multi-item scales would enhance measurement precision and allow for more nuanced assessment of user openness, including the distinction between preventive (maintenance) and reactive (rescue) use. Beyond the predictors examined here, future work should incorporate additional variables such as attitudes toward technology, personality traits, and prior exposure to digital therapy, which may further explain variance in willingness. Studies should also clarify how specific emotion regulation profiles and impulsivity dimensions shape engagement, and whether co-designed, gender-sensitive interventions can broaden appeal beyond young male gamers — particularly for women, older adults, and those less digitally inclined. Finally, recruitment beyond online crowdsourcing platforms, together with explicit examination of cultural and contextual factors (e.g., mental health stigma, local gaming norms), will be critical to ensure that digital mental health tools are both effective and equitable across diverse populations. Future research should explicitly examine how cultural context shapes willingness profiles and whether the predictors identified in this study operate similarly across different countries and cultural groups (Ellis et al., 2022; Alhassan et al., 2026).

## 5 Main contribution to the literature and conclusion

This study makes a significant contribution to the scientific literature by providing evidence from large-scale and cultural diverse sample that willingness to engage with game-based mental health interventions is both substantial and systematically heterogeneous. Moving beyond assumptions of uniform receptivity, it identifies distinct user profiles and demonstrates that openness is shaped by a complex interplay of socio-demographic, psychological, and behavioral factors, including mental health history, gaming habits, and emotion regulation processes. Notably, the findings challenge traditional “help-negation” assumptions by highlighting greater willingness among distressed individuals—particularly young men—and introduce a critical distinction between preventive and reactive engagement, offering a more precise framework for tailoring gamified mental health interventions.

Indeed, a key finding of the present study is that participants reported significantly lower willingness to engage with game-based interventions for the maintenance of well-being during periods of positive affect (i.e., preventive use) compared to their willingness to use such interventions to improve mental health during periods of distress (i.e., reactive use). This pattern underscores the need for deeper theoretical consideration, suggesting that preventive interventions—deployed in the absence of acute distress—may rely on distinct motivational mechanisms relative to reactive or “rescue” interventions, which are engaged in response to perceived psychological need.

Overall, the study shows that game-based and gamified app interventions are widely perceived as acceptable tools for supporting emotional regulation, mental health, and well-being. Higher receptivity among younger adults, males, frequent gamers, and individuals with prior mental health difficulties underscores the importance of user segmentation and targeted design. By clarifying who is most willing to engage and under what conditions, these findings advance the personalization of digital mental health solutions and contribute to bridging the gap between technological innovation and real-world impact in mental health care.

Finally, a substantial body of research on digital mental health technologies has primarily examined treatment adherence, usability, and user experience, with indicators such as engagement and perceived likability often used as indirect proxies for willingness to use these interventions (Baumel et al., 2019b; Torous et al., 2018b). In contrast, the present study extends this literature by directly assessing individuals' self-reported willingness, thereby providing a more explicit evaluation of user receptivity and complementing prior findings based on indirect behavioral and attitudinal measures.

### 5.1 Ethics approval and consent to participate

The study was carried out in accordance with the Declaration of Helsinki (WMA, 1964). Participants gave digital informed consent for their survey contribution. Participation was voluntary and restricted to those aged ≥18 years. All data was anonymously collected. Ethical approval for the research project (no. 8/18.11.2025) was obtained from the Ethics Committee of the Kazimierz Wielki University in Bydgoszcz.

## Consent for publication

No applicable.

## Funding

Chèque Inno Suisse 69099.1 INNO-ICT.

## Declaration of competing interest

All authors of the present manuscript have nothing to declare.

## Data Availability

The questionnaires and the data used in this study are available at Open Science Framework: https://osf.io/dwsjy/overview?view_only=475fbbd33b88428f9ed16ca332b537ee.
