# Supplementary material for: Willingness to use video-game or gamified application-based interventions to improve or strengthen emotional regulation, mental health, and mental well-being
Source: Internet Interv. 2026 May 1;44:100950. doi: 10.1016/j.invent.2026.100950 (PMC13136767; doi:10.1016/j.invent.2026.100950)
Supplement: Supplementary file 1 — Supplementary material [file mmc1.docx]

**Supplementary Material**

**Table S1.** Number of participants by country (48 countries from the all five continents)

| Frequencies of country_residence | | | | | | | |
| --- | --- | --- | --- | --- | --- | --- | --- |
| **country_residence** | | **Counts** | | **% of Total** | | **Cumulative %** | |
| Algeria |  | 1 |  | 0.0 % |  | 0.0 % |  |
| Australia |  | 67 |  | 1.8 % |  | 1.8 % |  |
| Austria |  | 7 |  | 0.2 % |  | 2.0 % |  |
| Belgium |  | 6 |  | 0.2 % |  | 2.2 % |  |
| Brazil |  | 6 |  | 0.2 % |  | 2.3 % |  |
| Canada |  | 181 |  | 4.8 % |  | 7.2 % |  |
| Chile |  | 47 |  | 1.3 % |  | 8.4 % |  |
| China |  | 1 |  | 0.0 % |  | 8.4 % |  |
| Croatia |  | 4 |  | 0.1 % |  | 8.5 % |  |
| Czech Republic |  | 9 |  | 0.2 % |  | 8.8 % |  |
| Denmark |  | 2 |  | 0.1 % |  | 8.9 % |  |
| Estonia |  | 8 |  | 0.2 % |  | 9.1 % |  |
| Finland |  | 6 |  | 0.2 % |  | 9.2 % |  |
| France |  | 41 |  | 1.1 % |  | 10.3 % |  |
| Germany |  | 81 |  | 2.2 % |  | 12.5 % |  |
| Greece |  | 23 |  | 0.6 % |  | 13.1 % |  |
| Hungary |  | 22 |  | 0.6 % |  | 13.7 % |  |
| India |  | 13 |  | 0.3 % |  | 14.0 % |  |
| Indonesia |  | 5 |  | 0.1 % |  | 14.2 % |  |
| Ireland |  | 14 |  | 0.4 % |  | 14.6 % |  |
| Israel |  | 11 |  | 0.3 % |  | 14.8 % |  |
| Italy |  | 81 |  | 2.2 % |  | 17.0 % |  |
| Japan |  | 5 |  | 0.1 % |  | 17.1 % |  |
| Kenya |  | 5 |  | 0.1 % |  | 17.3 % |  |
| Korea |  | 2 |  | 0.1 % |  | 17.3 % |  |
| Latvia |  | 6 |  | 0.2 % |  | 17.5 % |  |
| Malaysia |  | 1 |  | 0.0 % |  | 17.5 % |  |
| Mexico |  | 62 |  | 1.7 % |  | 19.2 % |  |
| Morocco |  | 1 |  | 0.0 % |  | 19.2 % |  |
| Netherlands |  | 33 |  | 0.9 % |  | 20.1 % |  |
| New Zealand |  | 15 |  | 0.4 % |  | 20.5 % |  |
| Norway |  | 4 |  | 0.1 % |  | 20.6 % |  |
| Philippines |  | 2 |  | 0.1 % |  | 20.6 % |  |
| Poland |  | 177 |  | 4.7 % |  | 25.4 % |  |
| Portugal |  | 106 |  | 2.8 % |  | 28.2 % |  |
| Romania |  | 1 |  | 0.0 % |  | 28.2 % |  |
| Saudi Arabia |  | 2 |  | 0.1 % |  | 28.3 % |  |
| Singapore |  | 1 |  | 0.0 % |  | 28.3 % |  |
| Slovakia |  | 4 |  | 0.1 % |  | 28.4 % |  |
| Slovenia |  | 6 |  | 0.2 % |  | 28.6 % |  |
| South Africa |  | 242 |  | 6.5 % |  | 35.0 % |  |
| Spain |  | 75 |  | 2.0 % |  | 37.0 % |  |
| Sweden |  | 14 |  | 0.4 % |  | 37.4 % |  |
| Switzerland |  | 1 |  | 0.0 % |  | 37.4 % |  |
| United Kingdom |  | 1235 |  | 33.0 % |  | 70.4 % |  |
| United Kingdom, United Kingdom |  | 1 |  | 0.0 % |  | 70.4 % |  |
| United States |  | 1103 |  | 29.5 % |  | 99.9 % |  |
| Vietnam |  | 4 |  | 0.1 % |  | 100.0 % |  |
|  | | | | | | | |

**Table S2.** Detailed descriptive statistics for all study variables

| Variable | N | Min-Max | Mean | SD | Skeweness* | Kurtosis* | Frequency |
| --- | --- | --- | --- | --- | --- | --- | --- |
|  |  |  |  |  |  |  |  |
|  |  |  |  |  |  |  |  |
| **Group of participants** |  |  |  |  |  |  | Gamers aged 18-25=1700(46.1 %);  Gamers aged 18-25 with depression and/or anxiety episodes=1312(35.6 %);  General population of non-gamers=673(18.3 %). |
| **Sociodemographics** |  |  |  |  |  |  |  |
| Age | 3745 | 18-79 | 30.94 | 12.76 | 1.49 | 1.42 |  |
| Sex |  |  |  |  |  |  | Male=1490(39.80%)  Female=2255(60.20%) |
| Country of residence | 3745 |  |  |  |  |  | UK=1236(33.0 %);  USA=1103(29.5 %);  South Africa=242(6.5 %);  Canada=181(4.8 %);  Poland=177(4.7 %);  42 Other countries=806(21.52%). |
| Employment status | 3745 |  |  |  |  |  | Full-time job=1258(39.3 %).  Part-time job=693(21.6 %) ;  Unemployed (and job seeking)=616(19.2 %) ;  Not in paid work (e.g. homework', 'retired or disabled) = 361(11.3 %);  Due to start a new job within the next month =55(1.7 %);   Other=220(6.9 %); |
| **Gaming frequency** |  |  |  |  |  |  |  |
| Daily hours playing video-game | 3060 | 2-5 | 3.64 | 1.16 | -.14 | -1.45 |  |
| Hours playing video-games (Weekdays) | 3685 | 0-120 | 13.50 | 12.26 | 2.25 | 8.69 |  |
| Hours playing video-games (Weekends) | 3742 | 0-60 | 7.93 | 6.31 | 2.10 | 8.15 |  |
| **Internet Gaming Disorder Test- 10 (IGDT-10)** |  |  |  |  |  |  |  |
| IGDT-Item1 | 3685 | 1-3 | 2.10 | .60 | -.05 | -.35 |  |
| IGDT-Item2 | 3685 | 1-3 | 1.62 | .63 | .52 | -.64 |  |
| IGDT-Item3 | 3685 | 1-3 | 1.84 | .72 | .25 | -1.06 |  |
| IGDT-Item4 | 3685 | 1-3 | 1.43 | .63 | 1.20 | .30 |  |
| IGDT-Item5 | 3685 | 1-3 | 1.65 | .70 | .61 | -.79 |  |
| IGDT-Item 6 | 3685 | 1-3 | 1.71 | .70 | .48 | -.91 |  |
| IGDT-Item 7 | 3685 | 1-3 | 1.32 | .58 | 1.64 | 1.60 |  |
| IGDT-Item 8 | 3685 | 1-3 | 2.21 | .70 | -.31 | -.96 |  |
| IGDT-Item 9 | 3685 | 1-3 | 1.11 | .36 | 3.66 | 13.46 |  |
| IGDT-Item 10 | 3685 | 1-3 | 1.34 | .56 | 1.45 | 1.13 |  |
| IGDT total mean score | 3685 | 1-3 | 1.63 | .39 | .53 | -.22 |  |
| **Impulsive Behavior Scale (UPPS-P)** |  |  |  |  |  |  |  |
| UPPS-P -Item1 (urgency) | 3678 | 1-5 | 2.35 | 1.01 | .73 | .09 |  |
| UPPS-P-Item2 (urgency) | 3678 | 1-5 | 2.52 | 1.08 | .52 | -.36 |  |
| UPPS-P mean for urgency | 3678 | 1-5 | 2.43 | .934 | .62 | -.043 |  |
| UPPS3-P-Item3 (premetitation) | 3678 | 1-5 | 2.52 | 1.00 | .12 | -1.08 |  |
| UPPS-P-Item4 (premeditation) | 3678 | 1-5 | 2.42 | .97 | .20 | -.94 |  |
| UPPS-P mean for premeditation | 3678 | 1-5 | 2.470 | .91 | .15 | -.89 |  |
| UPPS-P5-Item (preserverance) | 3678 | 1-5 | 2.77 | 1.06 | -.28 | -1.20 |  |
| UPPS-P6-Item (preserverance) | 3678 | 1-5 | 2.57 | 1.13 | -.05 | -1.40 |  |
| UPPS-P mean for preserverance | 3678 | 1-5 | 2.670 | .88 | -.15 | -.85 |  |
| UPPS-P7-Item7 (sensation seeking) | 3678 | 1-5 | 2.77 | 1.14 | -.36 | -1.30 |  |
| UPPS-P8 –Item8 (sensation seeking) | 3678 | 1-5 | 2.78 | 1.10 | -.36 | -1.22 |  |
| UPPS-P mean for sensation seeking | 3678 | 1-5 | 2.774 | 1.02 | -.36 | -1.04 |  |
| UPPS-P total mean score | 3678 | 1-5 | 2.90 | .52 | .36 | .29 |  |
| **Patient Health Questionnaire for Anxiety and Depression (PHQ-4)** |  |  |  |  |  |  |  |
| PHQ-Item1 (anxiety) | 3674 | 1-4 | 2.31 | .98 | .34 | -.86 |  |
| PHQ-Item2 (anxiety) | 3674 | 1-4 | 2.19 | 1.01 | .45 | -.88 |  |
| PHQ mean for anxiety | 3674 | 1-4 | 2.24 | .94 | .41 | -.81 |  |
| PHQ-Item3 (depression) | 3674 | 1-4 | 2.13 | .99 | .54 | -.74 |  |
| PHQ-Item4 (depression) | 3674 | 1-4 | 2.07 | .96 | .60 | -.58 |  |
| PHQ mean for depression | 3674 | 1-4 | 2.09 | .90 | .59 | -.56 |  |
| PHQ total mean score | 3674 | 1-4 | 2.17 | .85 | .47 | -.65 |  |
| **Difficulties in Emotion Regulation Scale Super Short Form (DERS-F-SSF-6)** |  |  |  |  |  |  |  |
| DERS-Item1 (awareness) | 3666 | 1-5 | 2.41 | 1.06 | .37 | -.85 |  |
| DERS-Item2 (clarity) | 3666 | 1-5 | 2.37 | 1.13 | .71 | -.30 |  |
| DERS-Item3 (goals) | 3666 | 1-5 | 3.29 | 1.22 | -.11 | -1.14 |  |
| DERS-Item4 (not acceptance) | 3666 | 1-5 | 2.56 | 1.28 | .47 | -.92 |  |
| DERS-Item5 (strategies) | 3666 | 1-5 | 2.32 | 1.14 | .71 | -.32 |  |
| DERS-Item6 (impulse) | 3666 | 1-5 | 1.95 | 1.07 | 1.11 | .52 |  |
| DERS total mean score | 3666 | 1-5 | 2.58 | .773 | .24 | -.52 |  |
| **Usefulness and willingness to use game/app/program to improve wellness** |  |  |  |  |  |  |  |
| Level of agreeing that a video-game, gamified app or Internet-based program could be useful for improving mental health | 3662 | 1-5 | 4.06 | .77 | -.92 | 1.78 |  |
| Willingness to use video game, app, and online-based interventions to develop or strengthen mental well-being? | 3661 | 1-4 | 3.35 | .70 | -.83 | .29 |  |
| Willingness to use video-game, app, and Internet-based interventions to develop or strengthen emotional regulation | 3663 | 1-4 | 3.25 | .75 | -.77 | .10 |  |
| Willingness to use video-game and Internet-based interventions to develop or strengthen mental health when feeling down | 3656 | 1-4 | 3.26 | .74 | -.77 | .24 |  |
| Willingness to use video-game or gamified apps-based interventions to maintain or strengthen mental well-being when feeling good | 3655 | 1-4 | 2.93 | .97 | -.96 | .87 |  |
| Type of games/apps/programs willing to try to improve emotional regulation, mental health and well-being | 3745 |  |  |  |  |  | 1=1249(34.2 %);  2=1147(31.4 %);  3=892(24.5 %);  4=360(9.9 %). |
| **Mental health problems** |  |  |  |  |  |  |  |
| Sought professional help for a mental health problem | 3745 |  |  |  |  |  | No=1748(47.7 %);  Yes=1916(52.3 %). |
| Diagnosed with a mental health problem | 3745 |  |  |  |  |  | No=1992(54.4 %);  Yes=1672(45.6 %). |
| Diagnosed substance use addiction | 3745 |  |  |  |  |  | No=3477(94.9 %);  Yes=188(5.1 %). |

Note. N = number of participants. Mix-Max = minimum-maximum. SD = standard deviation. *A general guideline for skewness is that if the number is greater than +1 or lower than –1, this is an indication of a substantially skewed distribution. For kurtosis, the general guideline is that if the number is greater than +1, the distribution is too peaked.

**Table S3.** The clusters centroids

|  | | | | | | | | | |
| --- | --- | --- | --- | --- | --- | --- | --- | --- | --- |
| **Clusters** | | **IER** | | **IMH** | | **SWBD** | | **SWBG** | |
| Cluster 1 |  | 3.898 |  | 3.941 |  | 3.857 |  | 3.680 |  |
| Cluster 2 |  | 2.766 |  | 2.896 |  | 2.808 |  | 2.369 |  |
|  | | | | | | | | | |

IER = willingness to use video-game apps to improve emotional regulation.; IMH = willingness to use video-game apps to improve mental health; SWBD = willingness to use video-game apps to improve mental wellbeing when feeling down; SWBD = willingness to use video-game apps to strength mental wellbeing when already feeling good.

**Figure S1.** Optimal number of clusters


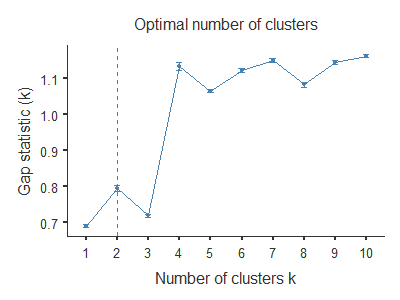


**Figure S2**. Cluster graphic


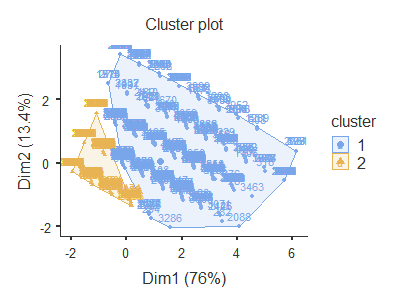


**Table S4.** Mean comparison between the two clusters: descriptive and inferential statistics

| Willingness measures | Clusters | N | M | SD | St. Error Mean | t | p-value | d | M-difference | St. Error difference |
| --- | --- | --- | --- | --- | --- | --- | --- | --- | --- | --- |
|  |  |  |  |  |  |  |  |  |  |  |
| IER | Cluster-1 | 1564 | 3.90 | .309 | .008 | 66.27 | <.001 | .510 | 1.132 | .017 |
|  | Cluster-2 | 2073 | 2.77 | .620 | .014 |  |  |  |  |  |
| IMH | Cluster-1 | 1564 | 3.94 | .239 | .006 | 64.92 | <.001 | .480 | 1.044 | .016 |
|  | Cluster-2 | 2073 | 2.90 | .601 | .013 |  |  |  |  |  |
| SWBD | Cluster-1 | 1564 | 3.86 | .365 | .009 | 59.13 | <.001 | .530 | 1.049 | .018 |
|  | Cluster-2 | 2073 | 2.81 | .626 | .014 |  |  |  |  |  |
| SWBG | Cluster-1 | 1564 | 3.68 | .515 | .013 | 53.88 | <.001 | .727 | 1.312 | .024 |
|  | Cluster-2 | 2073 | 2.37 | .853 | .019 |  |  |  |  |  |

N = number of participants; M = mean; SD = standard deviation; St. = standard; t = t-test of Student; d = Cohen’s d (measure of effect-size).

IER = willingness to use video-game and gamified apps-based interventions to improve emotional regulation.; IMH = willingness to use video-game and gamified apps-based interventions to improve mental health; SWBD = willingness to use video-game and gamified apps-based interventions to improve mental well-being when feeling down; SWBG = willingness to use video-game and gamified apps-based interventions to strength mental well-being when already feeling good.

**Table S5.** Sensitive analysis: results of the logistic regression without the two participants’ groups variables

| Model Coefficients - Clusters | | | | | | | | | | | | | | | |
| --- | --- | --- | --- | --- | --- | --- | --- | --- | --- | --- | --- | --- | --- | --- | --- |
|  | | | | | | | | | | | | **95% Confidence Interval** | | | |
| **Predictor** | | **Estimate** | | **SE** | | **Z** | | **p** | | **Odds ratio** | | **Lower** | | **Upper** | |
| Intercept |  | -0.93769 |  | 0.37060 |  | -2.5302 |  | 0.011 |  | 0.392 |  | 0.189 |  | 0.810 |  |
| Sexe |  |  |  |  |  |  |  |  |  |  |  |  |  |  |  |
| 2 – 1 |  | 0.43443 |  | 0.10548 |  | 4.1185 |  | < .001 |  | 1.544 |  | 1.256 |  | 1.899 |  |
| Employment status |  |  |  |  |  |  |  |  |  |  |  |  |  |  |  |
| 1 – 0 |  | -0.05553 |  | 0.17538 |  | -0.3166 |  | 0.752 |  | 0.946 |  | 0.671 |  | 1.334 |  |
| 2 – 0 |  | 0.18895 |  | 0.21185 |  | 0.8919 |  | 0.372 |  | 1.208 |  | 0.798 |  | 1.830 |  |
| 3 – 0 |  | 0.31522 |  | 0.33151 |  | 0.9509 |  | 0.342 |  | 1.371 |  | 0.716 |  | 2.625 |  |
| 4 – 0 |  | 0.18547 |  | 0.17493 |  | 1.0603 |  | 0.289 |  | 1.204 |  | 0.854 |  | 1.696 |  |
| 5 – 0 |  | 0.06478 |  | 0.16784 |  | 0.3859 |  | 0.700 |  | 1.067 |  | 0.768 |  | 1.483 |  |
| Diagnosed with mental health problem |  |  |  |  |  |  |  |  |  |  |  |  |  |  |  |
| 1 – 0 |  | 0.12358 |  | 0.09634 |  | 1.2828 |  | 0.200 |  | 1.132 |  | 0.937 |  | 1.367 |  |
| Diagnosed substance use addiction |  |  |  |  |  |  |  |  |  |  |  |  |  |  |  |
| 1 – 0 |  | 0.18940 |  | 0.18698 |  | 1.0130 |  | 0.311 |  | 1.209 |  | 0.838 |  | 1.743 |  |
| Age |  | 0.01197 |  | 0.00564 |  | 2.1246 |  | 0.034 |  | 1.012 |  | 1.001 |  | 1.023 |  |
| Daily hours playing video-game |  | -0.00551 |  | 0.03801 |  | -0.1450 |  | 0.885 |  | 0.995 |  | 0.923 |  | 1.071 |  |
| Weekly (Weekday?) hours playing video-game |  | 0.00920 |  | 0.00454 |  | 2.0239 |  | 0.043 |  | 1.009 |  | 1.000 |  | 1.018 |  |
| Week-end hours playing video-game |  | -6.32e−4 |  | 0.00855 |  | -0.0739 |  | 0.941 |  | 0.999 |  | 0.983 |  | 1.016 |  |
| IGDT total mean score |  | 0.56091 |  | 0.13054 |  | 4.2968 |  | < .001 |  | 1.752 |  | 1.357 |  | 2.263 |  |
| UPPS-P mean for urgency |  | 0.04586 |  | 0.05777 |  | 0.7939 |  | 0.427 |  | 1.047 |  | 0.935 |  | 1.172 |  |
| UPPS-P mean for lack premeditation |  | -0.09506 |  | 0.05226 |  | -1.8189 |  | 0.069 |  | 0.909 |  | 0.821 |  | 1.007 |  |
| UPPS-P mean for lack perseverance |  | -0.16484 |  | 0.04973 |  | -3.3148 |  | < .001 |  | 0.848 |  | 0.769 |  | 0.935 |  |
| UPPS-P mean for sensation seeking |  | -0.17572 |  | 0.04332 |  | -4.0561 |  | < .001 |  | 0.839 |  | 0.771 |  | 0.913 |  |
| PHQ mean for anxiety |  | 0.02055 |  | 0.06436 |  | 0.3193 |  | 0.750 |  | 1.021 |  | 0.900 |  | 1.158 |  |
| PHQ mean for depression |  | 0.0010 |  | 0.00500 |  | 2.0300 |  | 0.042 |  | 1.010 |  | 1.000 |  | 1.020 |  |
| DERS-Item1 (awareness) |  | -0.22533 |  | 0.04234 |  | -5.3219 |  | < .001 |  | 0.798 |  | 0.735 |  | 0.867 |  |
| DERS-Item2 (clarity) |  | 0.03248 |  | 0.04375 |  | 0.7423 |  | 0.458 |  | 1.033 |  | 0.948 |  | 1.126 |  |
| DERS-Item3 (goals) |  | 0.10365 |  | 0.04318 |  | 2.4005 |  | 0.016 |  | 1.109 |  | 1.019 |  | 1.207 |  |
| DERS-Item4 (not acceptance) |  | 0.11111 |  | 0.03847 |  | 2.8881 |  | 0.004 |  | 1.118 |  | 1.036 |  | 1.205 |  |
| DERS-Item5 (strategies) |  | -0.09875 |  | 0.04768 |  | -2.0711 |  | 0.038 |  | 0.906 |  | 0.825 |  | 0.995 |  |
| DERS-Item6 (impulse) |  | -0.00405 |  | 0.04794 |  | -0.0844 |  | 0.933 |  | 0.996 |  | 0.907 |  | 1.094 |  |
| Note. Estimates represent the log odds of "Clusters = 1" vs. "Clusters = 0" | | | | | | | | | | | | | | | |
|  | | | | | | | | | | | | | | | |
